# Exploring Choke Holds in Brazilian Jiujitsu Athletes: A Demographic Study

**DOI:** 10.7759/cureus.60618

**Published:** 2024-05-19

**Authors:** William B Harrington, Patrick R Fugler, Tatiana Midkiff, Stephen J Christensen, Eric Miller

**Affiliations:** 1 Medical School, Liberty University College of Osteopathic Medicine, Lynchburg, USA; 2 Emergency Medicine, Liberty University College of Osteopathic Medicine, Lynchburg, USA; 3 Physiology, Liberty University College of Osteopathic Medicine, Lynchburg, USA; 4 Medical School, Liberty College of Osteopathic Medicine, Lynchburg, USA; 5 Surgery, Centra Bedford Memorial Hospital, Bedford, USA; 6 Surgery, Liberty University College of Osteopathic Medicine, Lynchburg, USA

**Keywords:** brazilian jiujitsu, choke hold, cervical artery dissection, stroke, training demographics

## Abstract

Introduction

Brazilian jiujitsu is a relatively new sport that has grown exponentially in popularity along with the growth of the Ultimate Fighting Championship (UFC). In jiujitsu, there are a variety of submissions with a choke hold being one of the most popular. There is a subset of athletes in jiujitsu who believes chokes are safe. However, there have been case reports of relatively young athletes suffering strokes secondary to internal carotid or vertebral artery dissections after being placed in choke holds. There have been manuscripts describing the injury profile in jiujitsu, but none mention stroke or dissections. This study evaluated how frequently chokes happen in jiujitsu and if athletes have ever experienced symptoms consistent with cervical artery dissection (CAD). Additionally, this study aimed to describe the training frequency and baseline demographics of jiujitsu athletes.

Methods

A survey was distributed throughout social media platforms which asked both quantitative and qualitative questions regarding athlete training. The survey consisted of 28 questions which collected largely baseline grappling information about the participants such as how long they trained, how often they spar, favorite submission, how frequently they are choked, etc. This data was then analyzed using odds ratio and one sample t-test to evaluate for statistical differences.

Results

A total of 521 participants were included in the analysis. The participants were mostly male (84.7%), trained for four years, four times per week; 99.8% (520) participated in sparring, with an average age of 37; and 55.7% (290) have experienced symptoms consistent with CAD. Descriptive statistics revealed that individuals who were 37 years of age or younger were more likely to experience symptoms consistent with CAD (odds ratio: 1.5337 (95% confidence interval (CI): 1.0827-2.1727). Athletes that were 37 years of age or younger have been training for fewer years (4.7 years vs 8.8 years) but train more days per week (4.03 times per week vs 3.76 time per week), drill for a longer amount of time (46.8 minutes per class vs 38.3 minutes per class), attend longer classes (81.12 minutes vs 72.3 minutes), and train for a longer period of time per week (338.5 minutes vs 274.6 minutes) than athletes over 37 years. All previously mentioned variables were analyzed using a one sample t-test and were significant at the α = 0.05 level. The lone qualitative question regarding the term “train brain” revealed that of those who experienced it, 84.1% (58) described it as a cognitive/physical impairing event.

Conclusion

Jiujitsu athletes train multiple times per week and are frequently exposed to choke holds. There is no literature to examine the long-term effects of these chokes on the athlete’s cervical vasculature. Additional studies should be conducted to evaluate the effects of the repetitive stress placed on these vessels.

## Introduction

Brazilian jiujitsu is a relatively new sport that has increased substantially in popularity with the growth of the Ultimate Fighting Championships (UFC) from its launch in 1993 [1]. Jiujitsu is a sport which features a combination of traditional wrestling, judo, and submission grappling [2]. In a jiujitsu match, an individual wins one of four ways, opponent disqualification, submission, referee decision, or points [3]. The point system in jiujitsu is similar to collegiate wrestling (i.e., points for take downs, and various positions) [3]. If an athlete is placed in a submission, there are three possible outcomes: escape the submission, “tap out,” or suffer the ill effects of the submission [3]. By tapping out, the individual is acknowledging their opponent has beat them and want to stop the match prior to harm occurring [3]. As previously mentioned, if someone is placed in a submission and chooses not to tap, they can undergo serious bodily harm such as bone fractures, torn ligaments/tendons/muscles, unconsciousness, and rarely paralysis or death [2].

Similar to other martial arts, jiujitsu practitioners will practice multiple times per week with jiujitsu forums suggesting that athletes need to train three times a week [4]. Depending if the individual is a competitive jiujitsu athlete or trains as a hobby/self-defense, the number and intensity of their training sessions can greatly vary. A typical jiujitsu class will be structured with a warmup period, a drilling period, and a sparring period [5]. The drilling portion of the class is when the instructor will teach a specific technique and the students will repeatedly perform the movement for a certain amount of time. There is seemingly an infinite amount of techniques with one of the most popular techniques being a choke hold. Drilling most movements and submissions in jiujitsu is a rather benign activity; however, drilling choke holds may not be so safe. When a choke hold is placed in the drilling phase, the partner will usually tap out prior to unconsciousness, but individuals will occasionally be choked unconscious during a drilling session [6,7]. However, unlike in a match, the athlete will immediately be placed in another choke or submission after tapping. This is how submissions are drilled; one partner will be submitted repeatedly for either a set number of reps or time. This repetitive trauma could lead to dissection of the vessels in the neck which can manifest in a variety of different ways. Common symptoms from dissection of the carotid artery include ipsilateral neck pain, headache, facial or eye pain, and Horner syndrome with or without stroke symptoms [8]. Vertebral artery dissections typically present with lateral medullary syndromes, occipital headache, and neck pain [9]. There are case reports of individuals suffering either internal carotid, vertebral artery dissections, or strokes after drilling chokes during a routine training session [8,9].

As mentioned, the jiujitsu community is relatively new but is growing rapidly. A reddit group for jiujitsu holds 782,000 members and ranks in the top 1% of all reddit pages [10]. Unfortunately, there is a proportion of the jiujitsu community that believes chokes are completely safe [11]. Some individuals believe that if you do not tap to a choke hold, you will simply pass out and regain consciousness. Examples of this can be seen by celebrities/professional MMA fighters choking reporters unconscious, jiujitsu schools forcing their students to be choked unconscious as a right of passage, or popular podcasts speaking about the safety of chokes [12-14]. This nonchalant attitude carries over to competitive jiujitsu as well. Depending on the format of the jiujitsu tournament, athletes can be choked unconscious and allowed to compete again after a short 5-10-minute break [3]. Additionally, this rule set is applied to the youth who train jiujitsu as one of the largest jiujitsu organizations allows certain choke holds in children as young as four years old [3]. There are videos online of six-year-old children choking their opponent unconscious and gyms using these videos to promote their training curriculum [15].

With how new of a sport jiujitsu is, it is not well described in literature. There are several studies which describe the injury profile of jiujitsu, but none report the ill effects that can occur from choke holds (vascular dissections, stroke) [16,17]. This is a particular area of interest as internal carotid and vertebral artery dissections are the leading cause of stroke in individuals under the age of 40-45 [18]. For the purpose of this manuscript, we will collectively refer to internal carotid and vertebral artery dissections as cervical artery dissection (CAD). The training regimen of jiujitsu athletes is also not well described. To better understand jiujitsu, our study aimed to gather baseline demographic information regarding training habits, the prevalence of choke holds in jiujitsu, and if they have ever experienced symptoms compatible with a CAD. 

## Materials and methods

This study was approved and designated as exempt by the Liberty University’s Institutional Review Board (IRB). Data was collected by distributing a survey through social media platforms and groups. Qualtrics (Qualtrics, Provo, UT) was used for the development of the survey. After approval of the final survey, a weblink was generated which was posted to social media. This link was shared with an IRB-approved post to different social media platforms (Facebook, Instagram, etc.). Once the original post was created, it could then be shared freely across social media. The original IRB post ended up being shared to several large jiujitsu-specific Facebook groups. All surveys were completed online.

The survey consisted of 28 questions which captured both quantitative and qualitative data. The research questions and outcomes were developed using the experience of the authors with jiujitsu and interactions with the surveyed population through years of jiujitsu training. Questions which asked specifically about symptoms of CAD were formulated by finding common symptoms in medical literature and peer-reviewed articles. The study collected largely baseline grappling information about the participants such as how long they trained, how often they spar, favorite submission, how frequently they are choked, etc. The full survey is included in Appendix A. The survey did not collect identifiable information from participants, and duplicate prevention was conducted using tools in the Qualtrics software (IP address tracking and geographic location when completing the survey). The survey was built using branching and skip logic. The skip logic was also used to enforce the study’s inclusion/exclusion criteria. If an individual’s answer for a screening criteria did not meet eligibility criteria, they would be routed to the end of the survey. Participants who did not read/consent for the study, were under the age of 18, never trained jiujitsu, or had incomplete responses were not eligible for enrollment.

Once enrollment closed, the de-identified data was exported from Qualtrics into a CSV file. The data exported consisted of individuals who both met and did not meet eligibility criteria; therefore, all non-eligible participants were removed prior to data analysis. Data was then analyzed using Microsoft Excel (Microsoft Corporation, Redmond, Washington, United States) to calculate the mean and standard deviation for the variables included in this study. SAS OnDemand for Academics (SAS Institute Inc., Cary, NC, USA) was used to analyze differences in the study data using a one sample t-test. Differences were considered significant if p-value was less than 0.05. Variables were broken down into two groups, risk vs no-risk, or data above variable mean vs data below variable mean (i.e., participants who had been choked unconscious vs had not been choked unconscious or participant >37 years of age vs ≤37 years of age). Once participants were organized into their respective groups, each variable was analyzed to determine if any statistically significant relationships existed.

The survey consisted of one open-ended question in which the athlete described a jiujitsu term called train brain. As there was only one open ended question, there was not adequate data for formal qualitative analysis. However, the qualitative data was exported and analyzed in an Excel file. The data was coded independently by three authors and any inconsistencies between authors were revised. The qualitive data was analyzed using three themes, cognitive/physical impairment, pain, or cognitive/physical improvement.

## Results

The Qualtrics survey was distributed from July 2022 through August 2022 and yielded 573 responses. Out of the 573 responses, 51 participants did not meet eligibility criteria and were therefore excluded from the study. A total of 521 responses were included for analysis. Due to an error when publishing the survey, the question regarding gender was not published for the first 102 participants. The data from these participants were included in analysis as the primary endpoint for this study was to describe the training habits of jiujitsu athletes as a whole, not gender specific. This left a total of 419 participants who answered the gender question of which 355 (84.7%) were male. For the rest of the questions, all 521 participants answered. The average age of participants was 37.1 years and trained an average of four years. An average participant trained four times a week and would drill techniques for an average of 40 minutes per class. Ninety-nine percent (n = 520) of the study participants participated in sparring and sparred for an average of 30 minutes per class. A total of 412 (79.1%) participants reported being choked at least once per class, and 435 (83.4%) choked someone at least once per class. A total of 173 (33.2%) participants had been choked unconscious, while 189 (36.3%) participants had choked someone unconscious. A total of 290 (55.7%) participants experienced symptoms similar to CAD within 24 hours after training. Of those who experienced these symptoms, 170 (58.6%) experienced them less than 10 times. A full breakdown of the demographic information can be found in Table 1.

**Table 1:** Baseline training demographics for study participants

Characteristics	N (%)	Mean	Median
Age		37.1	37
Training length (years)		6.7	4
Sex (male)	355 (84.7%)		
Number of training sessions per week:		3.9	4
-Average time drilling per class (minutes)		43.3	40
Participates in sparring:	520 (99.8%)		
-Average time sparring per class (minutes)		34.4	30
Favorite submission:			
-Choke	312 (62.3%)		
-Upper extremity submission	155 (30.9%)		
-Lower extremity submission	28 (5.5%)		
-Other	6 (1.2%)		
Number of times submitted by a choke per class		1.7	1
Number of times submitting someone with a choke per class		3.1	2
Number of participants who have been choked unconscious	173 (33.2%)		
-If yes, how many times		3.4	2
Number of participants who has choked someone unconscious	189 (36.3%)		
-If yes, how many times		3.5	2
Number of participants with CVA dissection symptoms post training	290 (55.7%)		

**Figure 1:**
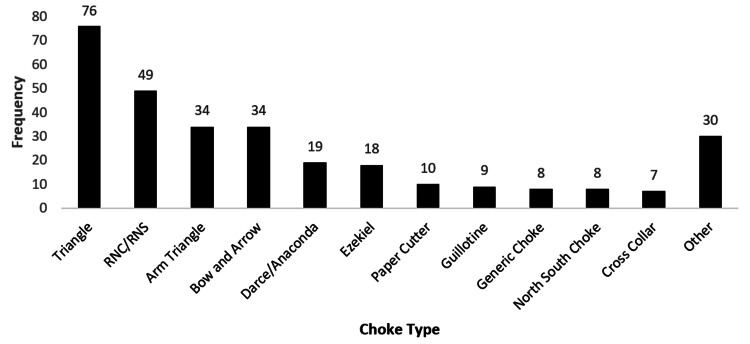
Favorite submission involving a choke hold Choke submission listed in other category: von flue (1), smother choke (1), side choke (1), peruvian necktie (3), mount smother (1), mandible choke (1), lapel/collar choke (5), gogoplata (2), clock choke (3), brabo choke (1), and baseball bat choke (5)

**Figure 2:**
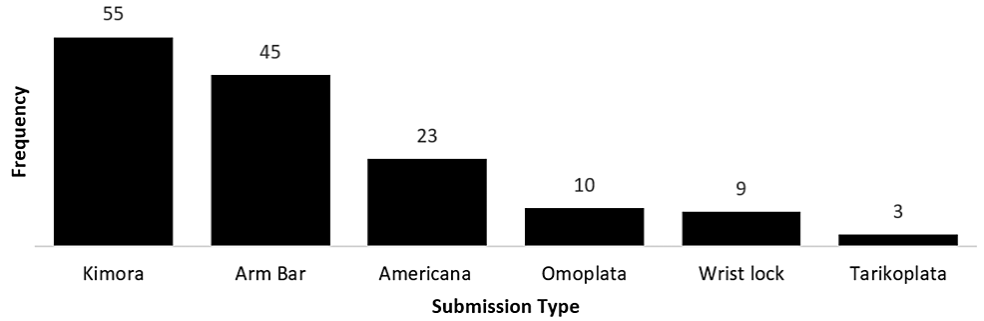
Favorite submission involving upper extremity

**Figure 3:**
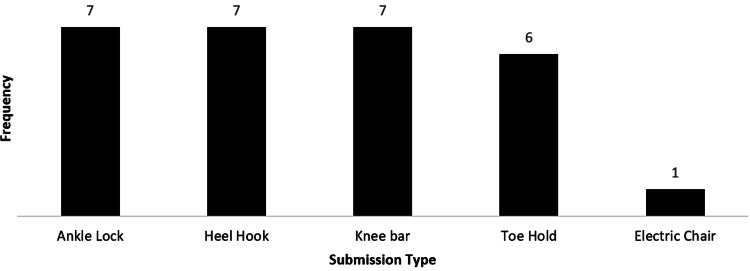
Favorite submission involving lower extremity

Moreover, 521 participants answered questions regarding their favorite submission. However, 20 participants submitted an answer which did not address the question. Out of the 501 participants included in this analysis, 312 (62.3%) indicated that a choke is their favorite submission. Upper extremity submissions were the second favorite submission with 155 (30.9%) participants. The full submission data is available in Figures 1-3.

Data was initially analyzed to determine if any of the variables differed in the number of participants who experienced symptoms consistent with CAD. To evaluate this, each variable was divided into two groups, less than or equal to the average or greater than the average (i.e., training length <= 4 years vs training length > 4). Once separated into their respective groups, odds ratios and 95% confidence intervals (CIs) were calculated in SAS. Age was the only variable which had a significant difference between groups. Participants who were 37 years of age or younger were 1.5337 (95% CI: 1.0827-2.1727) times the odds of a participant over 37 to experience symptoms consistent with CAD.

**Figure 4:**
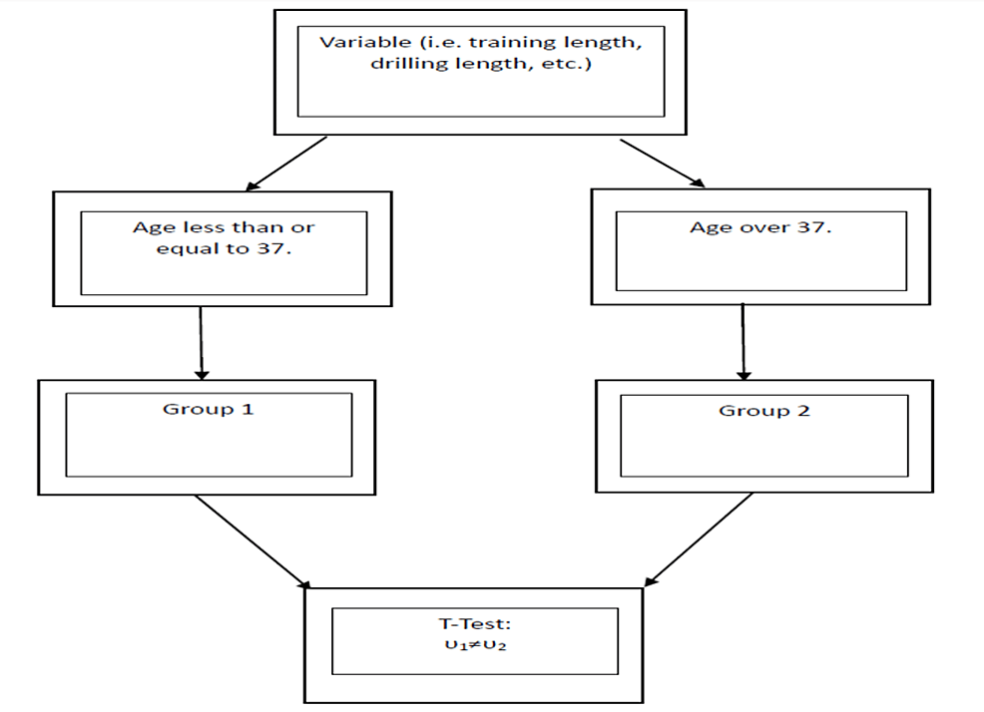
Flow chart which describes how the descriptive statistics were conducted

After revealing the significant difference based on age, additional analysis was conducted to evaluate potential causes for why younger athletes were more likely to experience symptoms. A one sample t-test was conducted to determine if there was a specific variable which significantly differed between age groups. Each variable was separated into two groups depending on age. A flow chart describing how each variable was categorized can be found in Figure 4. Two new variables were created by combing data from two existing variables. The new variables were training time per class (drilling time + sparring time) and time training per week (training days per week + training time per class).

Five variables were found to have differed significantly based on age: how long they have been training (p-value = <0.0001), number of training sessions per week (p-value = 0.0177), time drilling per class (p-value = 0.0009), training time per class (p-value = 0.005), and time training per week (p-value = 0.0001). Athletes that were 37 years of age or younger have been training for fewer years (4.7 years vs 8.8 years) but train more days per week (4.03 times per week vs 3.76 time per week), drill for a longer amount of time (46.8 minutes per class vs 38.3 minutes per class), attend longer classes (81.12 minutes vs 72.3 minutes), and train for a longer period of time per week (338.5 minutes vs 274.6 minutes). While not statistically significant individuals, 37 or younger sparred for longer each class (35.7 minutes per class vs 33 minutes per class). A full review of descriptive statistics can be found in Table 2.

**Table 2:** Descriptive statistics to determine if there may be a reason why younger individuals were more likely to experience symptoms consistent with cervical artery dissection

	t value	p-value	Group 1 mean (stvd)	Group 2 mean (stvd)
Had been choked unconscious vs not	-1.24	0.2162	31% (0.46)	36% (0.48)
Choked someone unconscious vs not	-1.48	0.1386	33% (0.47)	40% (0.49)
Training length (years)	-7.56	<0.0001	4.7 years (4.1)	8.9 years (8.0)
Number of training sessions per week	2.38	0.0177	4.0 (1.38)	3.8 (1.3)
Length of drilling	3.35	0.0009	46.9 min (30.3)	39.3 min (19.4)
Length of sparring	1.48	0.1390	35.4 min (16.4)	33.3 min (15.9)
Favorite submission is a choke	0.14	0.8913	62.8% (0.48)	62.2% (0.49)
Average class length	3.50	0.0005	81.12 min (36.1)	72.3 min (26.4)
Average time training per week	3.90	0.0001	338.5 min (217.3)	274.6 min (145.5)

Qualitative data

In jiujitsu, athletes may experience fogginess, forgetfulness, headaches, etc. after a training session. In some gyms, this term has been referred to as “train brain.” This term does not appear in a literature search. Therefore, a question was developed to ask individuals who experienced train brain to describe it. There was a total of 77 participants who answered they had experienced train brain. Of the 74 responses, 69 were used for analysis, five responses were excluded from analysis due to lack of information. As previously mentioned, since there was only one question, there was not adequate data for formal qualitative analysis. However, the information from the open-ended question was independently coded by three of the authors. From these codes, three themes were created and applied to the responses. Any discrepancies were discussed by the authors, and if a conscious could not be achieved, the data was omitted. The themes were cognitive/physical impairment, cognitive/physical improvement, and pain. Fifty-eight participants were assigned to the cognitive/physical impairment theme. Several examples from this theme are “bit foggy, hard to find words after training, a little light headed,” “disoriented,” “can’t think, put together sentences," just foggy and tired,” “fogginess, trouble "finding words," trouble concentrating, headache,” along with others. Ten participants were assigned to the cognitive/physical improvement theme. Several examples from this theme are "when you focus only on training and nothing else,” “when everything clicked and made sense. You finish training exhausted but emotionally high,” “endorphins from the workout feel so good that all anxieties are gone for a bit. Even though the body is sore, there is a sense of calm and achievement in the brain,” along with others. Lastly, one participant was assigned to the pain them. Several of their responses were “headaches, neck pain.” All of the qualitative data responses can be found in Appendix B. The qualitative data showed that out of those participants who experienced train brain, 84.1% described it as an impairing event.

## Discussion

Given the rapid increase in the popularity of jiujitsu, there is little literature to suggest the physiological effects of choke holds. There are several manuscripts which describe the injury profile of jiujitsu. However, none of them describe internal carotid or vertebral artery dissection [16,17]. CAD is the most common causes of stroke in individuals under 45, with 25% of ischemic strokes being caused by CAD [19]. However, this number may be underreported due to difficulty with diagnosis and potential transient nature [20]. Symptoms consistent with CAD such as dizziness, headache, and neck pain were reported by 55% of the participants. While it is highly unlikely that each of 290 participants who experienced these symptoms suffered CAD, it does raise the question about potential cervical artery disease in jiujitsu athletes. Without clinical workup, we cannot conclude what caused their symptoms. This study did not ask if any of the participants received care for their symptoms. Due to the potential transient nature of vascular injury, jiujitsu athletes may not seek care and attribute their symptoms to more common reasons such as dehydration or trauma [21]. Along with dehydration, other less severe causes of these symptoms could be simple ischemia, vasospasm, microemboli, transient global amnesia (TGA), concussion, and others. There is no literature to suggest the uninsurance rate in jiujitsu athletes. Likewise, most athletes work a job to help fund their training and competitions, making it hard to decipher the average income for jiujitsu athletes. Potentially due to jiujitsu being a combat sport, lack of health insurance, distrust of the medical system, or financial strain, some athletes tend to only seek medical care if it is absolutely necessary [21]. Even if an athlete presented to the emergency room for these symptoms, one in 30 athletes will be misdiagnosed [20].

As previously mentioned, there have been several case reports and one case series of jiujitsu athletes suffering CAD after training [8]. While the patient demographics differed between the cases, each participant reported being in a choke hold prior to their symptom onset [8]. There is literature which suggests that there is an association between sportive chokes and CAD [22]. As a choke hold is placed, whether in drilling or in sparring, it will compress the internal carotid arteries leading to decreased blood flow to the middle cerebral artery and eventual unconsciousness [23]. The compression of the carotid arteries can lead a pressure of 400 mmHg over the carotid bifurcation [23]. If a choke hold is placed correctly, meaning large occlusion of internal carotid arteries, the flow reaches a minimum flow rate in the internal carotid artery and middle cerebral arteries 1.3 and 9.5 seconds, respectively [23]. Simply put, a well-placed choke places a large amount of stress to the internal carotid arteries.

Although there is a large amount of stress placed on the vessels with a well-placed choke, the duration of the choke is relatively short lived as the average time to unconsciousness is nine seconds [23]. An area of concern are chokes that are not placed in the right anatomical position. If a choke is not placed over the correct anatomical position, it would decreased the compression of the internal carotids leading to a choke which is inadequate for unconsciousness. This is more common in an amateur mixed martial arts and regional jiujitsu tournaments, but even in professional fighting, there have been instances of athletes being in chokes for 30 seconds or longer [24]. There are instances of jiujitsu practitioners reporting being in choke holds for upward of three minutes without tapping [25]. Athletes will continue to hold the submission in the hope their opponent will eventually tap or pass out [25]. The partial compression of the carotid vessels could lead to high velocity turbulent flow in those vessels. This could be problematic as turbulent flow in the carotids could cause plaque dislodgement leading to embolic stroke. Additionally, atherosclerotic disease of the internal carotid arteries is a risk factor for dissection without the added stress of a choke [19]. Rarely, if the pressure is placed directly to the carotid bodies, it can cause cardiac dysrhythmias leading to cardiac arrest and death [26].

Compression of the blood vessels allows for the accumulation of vasodilatory metabolites which when released cause an immediate dilation of the vasculature. While this dilation is not affected by the duration of the compressive forces, it does increase when there are repetitive compressive forces are applied [27]. Our study showed that athletes are subjected repetitive compressive forces multiple times per class during the drilling portion and during the sparring portion. As athletes train on average 3.9 times per week, they are subjected to these vasodilatory effects often. There is no literature which evaluates vascular changes in jiujitsu athletes during a training session. There have been studies which evaluated the physiology of choke holds, but none have been in jiujitsu athletes [28].

Due to the location of the vasculature in the neck, the internal jugular vein would be compressed along with the internal carotid. As it takes less pressure to compress a vein than an artery, the internal jugular vein would compress prior to the internal carotid artery. Our data showed that jiujitsu athletes choked on average 1.7 times per class and trained an average of 3.9 classes per week. This would minimally lead to partial/total internal jugular and internal carotid occlusion 6.6 times per week. Our average participant could have been choked an estimated 1,414.4 times throughout their jiujitsu career. However, our data asked specifically how many time athletes were submitted per class. This would not account for being placed in chokes during the drilling portion of the class or ill-placed chokes they may have escaped during sparring. Meaning our data likely underreports the total amount of times an athlete has been choked. Over time, the repetitive stress placed from chokes could cause damage to the vessel wall leading to stenosis or venous insufficiency. The latter of which has been associated with episodes of TGA with strenuous activity [29]. TGA is defined as an acute onset of temporary anterograde amnesia and can be precipitated by the aforementioned strenuous activity or choking incidents [30]. Symptoms of TGA may consist of disorientation, forgetfulness, or inability to retain new information [30,31]. Our qualitative data revealed that some participants experienced forgetfulness, disorientation, and the inability to retain new information after a hard training session.

There are certain movements in jiujitsu which lead to a large amount of pressure on the cervical and thoracic spine. Over time, the cumulative effects of this pressure can lead to degenerative disc disease and cervical spine injury [32]. Additionally, there are rare instances of serious cervical spine injury during training, such as subluxation of a vertebral body [33]. The added stress to the cervical spine may lead to osteophyte formation, disc herniation, or loss of vertebral height. All of which can lead to displacement of the vertebral arteries. In addition to the increased risk of stroke, spondylosis increases the risk of vertebral artery dissection in the general population, which may compound the risk to jiujitsu athletes [33]. Displacement of the vertebral arteries leads to a change in blood flow leading to an increased risk of posterior circulation (PC) stroke [34]. Dissections of the vertebral arteries typically occur due to manipulation, trauma, or positional (hyperextension or stretching) of the neck [22]. Neck injuries are common in jiujitsu which would increase the risk of vertebral artery dissection in athletes [18]. There are currently no studies which evaluate the long-term effects this stressor has on vessel walls or the cardiovascular system.

Limitations

Due to oversight, for the first 102 respondents of the survey, we did not collect gender information nor did we ask if participant sought care for their symptoms. The goal of this study was to determine how frequently chokes occur in the sport of jiujitsu and the question was originally omitted. Once this oversight was noticed, the survey was updated with a gender question. As previously mentioned, the goal of this study was to explore choke holds, and therefore, this did not impact the integrity of the study. This survey asked athletes to describe their training regiment which may have led to potential recall bias from the participants. Another possible limitation associated with our study is that the familywise error rate across analysis was not controlled. We did not believe the “universal null hypothesis” for the two groups applied to our particular study. Additionally, we consider this research to be preliminary and encourage further replication.

## Conclusions

Our study collected training demographics to give better insight on how jiujitsu athletes train. The data revealed that athletes train an average of four times a week with an average class length of 77.7 minutes (34.4-minute sparring, 43.3-minute drilling) for an average of four years. During a typical week of training, our individuals were choked an average of 1.7 times per class and choked someone an average of 3.4 times per class. Meaning, the average participant in our study has been choked an estimated 1,414.4 and choked someone 2,828.8 times throughout their jiujitsu career. Given the fact that our study population is young (37 years of age), they will have years of stress placed on their cervical vasculature. Our study showed that athletes who were 37 years or younger had a 1.5337 times the odds of experiencing symptoms similar to CAD than those older than 37. Our analysis showed athletes that were 37 years of age or younger have been training for fewer years (4.7 years vs 8.8 years) but train more days per week (4.03 times per week vs 3.76 time per week), drill for a longer amount of time (46.8 minutes per class vs 38.3 minutes per class), attend longer classes (81.12 minutes vs 72.3 minutes), and train for a longer period of time per week (338.5 minutes vs 274.6 minutes) than those older than 37. Similarly, 55.7% of our study participants had experienced symptoms consistent with a cervical artery dissection. While unlikely the 290 participants experienced dissection, it does raise the question about potential cervical vessel disease. The long-term effect of this added stress should be explored. Future studies should be aimed at using imagining techniques to evaluate for potential stenosis, venous valve insufficiency, arteriosclerosis, and mapping of the cervical vasculature.

## Appendices

Appendix A

Start of block: Default question block

Q1 Please download the consent form, then click the next button

Q2 Have you downloaded the consent form?

          o Yes, and I agree to participate in the study

          o I do not want to participate in the study

Skip to: End of survey if have you downloaded the consent form? = I do not want to participate in the study

Q3 How old are you? Skip to: End of survey if condition: Click to write the question... Is less than 18. Skip to: End of survey.

Q4 Do you currently train or have ever trained Brazilian jiujitsu or mixed martial arts (MMA)?

           o Yes

           o No

Skip to: End of survey if do you currently train or have ever trained Brazilian jiujitsu or mixed martial arts (MMA)? = No

Q5 How long have you trained (in years)?

Q6 What gender do you identify as?

          o Male

          o Female

          o I'd rather not say

Q7 How many times per week do you train (days)?

Q8 On an average day while training, how long do you drill techniques (minutes)?

Q9 Do you participate in rolling (sparring)?

            o Yes

            o No

Display this question: If do you participate in rolling (sparring)? = yes

Q10 On average, how long do you roll (for overall minutes) per class?

Q11 What is your favorite submission?

Q12 How often per class do you get submitted with a choke?

Q13 How often per class do you submit someone with a choke?

Q14 Have you ever been choked unconscious "blacked out/choked out"?

           o Yes

           o No

Display this Question: If have you ever been choked unconscious "blacked out/choked out"? = yes

Q15 How many times have you been choked out?

Q16 Have you ever choked someone unconscious (blacked out/choked out)?

         o Yes

         o No

Display this Question: If have you ever choked someone unconscious (blacked out/choked out)? = yes

Q17 How many times have you choked someone unconscious?

Q18 After training (whether immediately or a day later), have you ever felt lightheaded, dizzy, or nauseous, have vomited, or had a headache, vision problems, or neck pain?

         o Yes

         o No

Display this question:

If after training (whether immediately or a day later), have you ever felt lightheaded, dizzy, nausea... = yes

Q19 How many times have you felt this way?

        o <10

        o <25

        o <50

        o <100

        o I have no idea, but it happens a lot

        o It happens almost every class

Q20 Have you ever experienced "train brain"

        o Yes

        o No

Display this question: If have you ever experienced "train brain" = Yes

Q21 How would you described train brain?

Appendix B

**Table 3:** Appendix B

You feel like you don’t think, just going through the motions after training	Cognitive/physical impairment
Headaches, neck pain	Pain
Difficulty concentrating	Cognitive/physical impairment
Generally disassociated from reality for a brief time. Could also be the time so it's hard to say.	Cognitive/physical impairment
Mental exhaustion. Slow function	Cognitive/physical impairment
Surreal	Cognitive/physical impairment
Foggy, tired, lethargic	Cognitive/physical impairment
Mentally exhausted	Cognitive/physical impairment
Subconsciously thinking of jiujitsu or jiujitsu-related topics	Cognitive/physical impairment
A mental overload where I can’t process any new information for an hour or so	Cognitive/physical impairment
Tired, fatigue, unclear	Cognitive/physical impairment
Overexhaustion	Cognitive/physical impairment
Feels dead	Cognitive/physical impairment
Fog	Cognitive/physical impairment
A foggy, almost droning sensation	Cognitive/physical impairment
Sluggish	Cognitive/physical impairment
Haziness	Cognitive/physical impairment
Hazy	Cognitive/physical impairment
Not sure	
Unable to focus on other subjects	Cognitive/physical impairment
Feeling punch drunk or fuzzy	Cognitive/physical impairment
It’s kind of like the ability to order your thoughts in a way you can then carry out a task. Like I need to go to the shops, get shoes on, get wallet, get car keys… wait what do I need car keys for? Ahh go to shop..	Cognitive/physical impairment
When everything clicked and made sense. You finish training exhausted but emotionally high.	Cognitive/physical impairment
When you focus only on training and nothing else	Cognitive/physical impairment
Foggy and tired	Cognitive/physical impairment
3	
Fuzzy headed, tired	Cognitive/physical impairment
Sometimes too much info to take in to where I cant recall anything	Cognitive/physical impairment
Hard to describe. Headache, euphoria, brain fog	Cognitive/physical impairment
Groggy feeling after training	Cognitive/physical impairment
Lack of focus/forgetful. ADHD doesn't help.	Cognitive/physical impairment
"High"	Cognitive/physical impairment
Disoriented	Cognitive/physical impairment
Brain fog, exhaustion	Cognitive/physical impairment
Endorphins from the workout feel so good that all anxieties are gone for a bit. Even though the body is sore, there is a sense of calm and achievement in the brain.	Cognitive/physical impairment
Foggy or like in a haze. Can't grab the concept of technique	Cognitive/physical impairment
Foggy headed	Cognitive/physical impairment
Little foggy, easily distracted	Cognitive/physical impairment
Not able to retain the information given during a class	Cognitive/physical impairment
Mental exhaustion after class	Cognitive/physical impairment
Bit foggy, hard to find words after training, a little lightheaded	Cognitive/physical impairment
Feeling of clarity of focus	Cognitive/physical impairment
Mental fatigue	Cognitive/physical impairment
Being on auto pilot	Cognitive/physical impairment
Not focused on the instruction	Cognitive/physical impairment
I assumed train brain was when you only think about your time on the way while in class. It’s a natural and purely survival feeling	
Obsession	Cognitive/physical impairment
Always thinking about training	Cognitive/physical impairment
I can't stop thinking about techniques	Cognitive/physical impairment
Like being drunk	Cognitive/physical impairment
Fuzzy brain, inability to focus/concentrate. Moment of vagueness	Cognitive/physical impairment
When I keep thinking of how to train techniques together and cannot stop	Cognitive/physical impairment
Like having gotten a lot of fresh air, feeling kind of exhausted but also very relaxed	Cognitive/physical impairment
Tiredness. Difficulty focusing	Cognitive/physical impairment
Always thinking of training. Had to look up the definition on Google though as I have never heard of this	Cognitive/physical impairment
Fatigue, exhaustion, anxiety	Cognitive/physical impairment
Lightheaded. One time I was disoriented.	Cognitive/physical impairment
Brain fog after training. Different from just being tired, it's closer to being dazed. I notice water and electrolytes helps minimize it.	Cognitive/physical impairment
Foggy	Cognitive/physical impairment
Complete exhaustion, brain turned off	Cognitive/physical impairment
No idea	
Fogginess, trouble "finding words," trouble concentrating, headache	Cognitive/physical impairment
Senses are dull, tired	Cognitive/physical impairment
Can’t get my back off the mat and get beat on	Cognitive/physical impairment
More of a burnt out feeling like MMA and working out is a chore I have to do instead of something I look forward to	Cognitive/physical impairment
Good	Cognitive/physical impairment
1000-yard state, mental “numbness” post rolls	Cognitive/physical impairment
It’s like a head fog that doesn’t make u feel bad but almost like there’s a film on a movie screen and it’s in the first person	Cognitive/physical impairment
Dizzy	Cognitive/physical impairment
Really slow thinking	Cognitive/physical impairment
Forgetting what I'm trying to say half way through saying it, or what I'm trying to do	Cognitive/physical impairment
Just over training to the point of no longer consuming any information or desire to consume any	Cognitive/physical impairment
Can’t think, put together sentences. Just foggy and tired	Cognitive/physical impairment
Fog	Cognitive/physical impairment
